# Relationship between cortisol, Interleukin-6 and homocysteine in Alzheimer's disease

**DOI:** 10.5339/qmj.2021.33

**Published:** 2021-09-06

**Authors:** Neelam Yeram, Shubhangi Dalvi, Ranjit Mankeshwar, Vinayak Patil, Vinayak Kale, Kamlesh Jagiasi, Leela Abichandani

**Affiliations:** ^1^Department of Biochemistry, Grant Government Medical College & Sir J. J. Group of Hospitals, Mumbai, India E-mail: shubhangigg03@gmail.com; ^2^Grant Government Medical College & Sir J. J. Group of Hospitals, Mumbai, India; ^3^Department of Psychiatry, GGMC & Sir J. J. Group of Hospitals, Mumbai, India; ^4^Department of Neurology, GGMC & Sir J. J. Group of Hospitals, Mumbai, India; ^5^Department of Biochemistry, Vedantaa Institute of Medical Sciences, Dahanu, India; ^6^Department of Community Medicine, Grant Government Medical College & Sir J. J. Group of Hospitals, Mumbai, India

**Keywords:** cortisol, Interleukin-6, homocysteine, Alzheimer's disease

## Abstract

Objective: Alzheimer's disease (AD) is characterised by progressive cognitive decline due to neurodegeneration. Over activation of the hypothalamic–pituitary–adrenal axis, oxidative stress and inflammation potentially damage the neuronal system, affecting cognition.

Aim: This study aimed to assess the relationship between serum cortisol, Interleukin-6 (IL-6) and homocysteine (Hcy) levels in AD.

Methods: Case-Control observational study consisting of 71 patients with AD and 70 healthy controls above 60 years of age. Serum samples were analysed for cortisol, IL-6 and Hcy levels using chemiluminescence immunoassay (Immulite 1000) technique. Cognitive functions were measured using the Mini-Mental State Examination (MMSE) Score. AD subjects were categorised based on the modified Kuppuswamy socioeconomic status scale. Statistical evaluation was conducted using SPSS Statistics software. Group data were analysed using a two-tailed Student's t-test, analysis of variance (ANOVA), the Mann–Whitney U test and Pearson's correlation test.

Results: Serum cortisol, IL-6 and Hcy levels were significantly increased (*p* < 0.01) in AD (cortisol: 19.69 ± 8.96 ug/dl; IL-6: 10.27 ± 2.76 pg/ml; Hcy: 23.29 ± 3.81 μmol/l), as compared with the controls (cortisol: 13.37 ± 5.59 ug/dl; IL-6: 3.37 ± 0.79 pg/ml; Hcy: 8.25 ± 2.36 μmol/l). MMSE scores in AD were negatively correlated with cortisol, IL-6 and Hcy levels.

Conclusions: Serum cortisol, IL-6 and Hcy levels are independent biomarkers for AD progression. Hypercortisolaemia, hyperhomocysteinemia and inflammation play important roles in AD-related cognitive dysfunction and are interlinked.

## Introduction

Today, one of the most commonly occurring neurodegenerative diseases in the elderly is dementia, specifically Alzheimer's disease (AD). AD is generally characterised by the extensive degeneration of neuronal tissue resulting in progressive impairments in cognitive functions and behaviour. One of the most frequent psychiatric complications affecting 50% of patients with AD is stress and depression, which potentially activates the hypothalamic–pituitary–adrenal axis. Over activation of this metabolic axis has been reported to potentially lead to neuronal damage, neuroinflammation, hippocampal atrophy and cognitive impairment, and an increased cortisol level has been associated with more rapid disease progression in patients with AD.^1,2^

A glucocorticoid hormone, cortisol is secreted by the cortex regions of the adrenal glands in association with stress responses and mediates metabolic processes such as energy mobilisation, cerebral perfusion, cardiovascular output enhancement, blood flow redistribution and immune system modulation.^3^ Evidence has shown that the excess production of glucocorticoids in the system leads to the enhanced aggregation and deposition of amyloid-beta proteins in the brain tissues of patients with AD. The blood-brain barrier is found to be permeable to cortisol, thereby allowing the entry of cortisol molecules into the neuronal tissue, where it potentially binds to its receptors located in the specific brain regions related to cognition, learning and memory, such as the amygdala, hippocampus and frontal lobes. The chronic increase in the concentrations of cortisol is found to be markedly associated with hypertension and cerebral atrophy.^3,4^ Fewer studies have shown that chronic stress also has an impact on the levels of cytokines, including Interleukin-6 (IL-6) that represent the extent of neuroinflammation, which is a pathological hallmark of AD.^5^ IL-6 is a multifunctional cytokine from the neuropoietin family of cytokines. It is a major regulator of the inflammatory response and neurotrophically affects the neurons in both direct and indirect ways.^6^

Studies have also found that elevated homocysteine (Hcy) concentrations, i.e. hyperhomocysteinemia, remarkably damages the brain hippocampus and surrounding regions, which is seen as the proximal cause of memory deficits. Hcy is considered to be associated significantly with AD after carotid atherosclerosis.^7^ It is an amino acid that contains sulphur and is produced by a methionine methylation reaction. Excess concentrations of methionine promote the trans-sulphuration pathway, which results in the conversion of Hcy into cysteine. The end product of this pathway, glutathione, is a strong antioxidant, which protects the body cells and its components against damage caused by oxidative stress. Secondly, the accumulation of Hcy also leads to the process of Hcy remethylation into methionine, which uses methylcobalamin and folic acid as potential cofactors. Elevated Hcy levels due to the deficiency of B vitamins in AD promotes oxidative stress and markedly enhances neuronal damage.^8-10^ Lack of proper dietary nutrition, a low education level and the lifestyle of individuals throughout their life span are known to potentially affect the occurrence of AD, which ultimately depends on socioeconomic status (SES). Moreover, biological and psychological stress varies considerably between males and females; therefore, it would be instructive to compare the variability in study factors with regards to gender.^11,12^

Since AD is a multifactorial disease, there is a crucial need to understand the interactions among the factors that are influenced and are disrupted in this complex disease. Associations between the proinflammatory cytokine IL-6 and homocysteine toxicity along with the stress marker cortisol would offer a broad vision for speculations involving these markers in AD. This study aimed to assess the relationship between serum cortisol, IL-6 and Hcy levels with the cognitive functions measured by the Mini-Mental State Examination (MMSE) Score in patients with AD. Neurologically healthy subjects’ age- and sex-matched to the AD cases were recruited as a control group for comparison purposes.

## Methods

### Sample Analysis

The study consisted of 71 cases of clinically diagnosed AD subjects (AD group) and 70 healthy individuals (Control group) of both sexes above 60 years of age. Individuals visiting the neurology outpatient department (OPD) or admitted in the general government hospital, located in a metropolitan city of the western region of India were considered for screening. AD subjects were diagnosed using the National Institute of Ageing and the Alzheimer's Association (NIA-AA) criteria by clinicians of the neurology department. AD subjects were involved in this study irrespective of their disease stage, with durations of symptoms ranging from 6 months to 5 years. Subjects suffering from chronic diabetes, chronic alcoholism, cerebral stroke, head injury, poisoning, schizophrenia or any other mental illness except AD were excluded from the study. Informed consent was obtained from participants or their representatives (in the case of AD subjects). The study received ethical approval by the institutional ethics committee of the hospital (IEC/Pharm/328/15, dated 26-08-2015). Blood samples of all participants were collected in plain tubes; serum was separated by centrifugation and stored at − 80° in a deep freezer. Analysis of serum samples for levels of cortisol, IL-6 and Hcy was carried out by chemiluminescence immunoassay (Immulite 1000). Cognitive functions were measured using the MMSE Score. Subjects were classified by socioeconomic status (SES) according to the modified Kuppuswamy socioeconomic status scale.^10,13^

### Statistical Analysis

SPSS statistics software (version 25) was used for statistical evaluation. Descriptive statistics were conducted to obtain results which were represented as mean ±  Standard Deviation. Differences between group means were compared using a two-tailed Student's t-test or analysis of variance with a 95% confidence interval and alpha of 0.05. The differences between the values of variables in the gender and SES categories were assessed using the Mann–Whitney U test. Relationships between categorical variables were accessed by a chi-square test with a 95% confidence interval. The relationships between study parameters in the AD and control groups were examined using Pearson's correlation. Differences were considered as statistically significant at *p* < 0.05, and *p* < 0.01 was considered as highly significant. Schematic representation of the data was performed using linear regression graphs.

## Results

The AD group has significantly higher MMSE scores than the control group. The cortisol, IL-6 and Hcy levels were significantly higher in the AD group than in the control group (Table 1).

The control group consisted of 40 males (57.14%) and 30 females (42.86%), while the AD group consisted of 46 males (64.79%) and 25 females (35.71%). The chi-square test showed a nonsignificant relationship (*p* = 0.132). In the control group, male subjects had a significantly higher mean age than females. No significant difference was seen in any other variable of the control and AD groups.

The majority of individuals in the control and AD groups belonged to the middle class of SES. However, no significant (*p* = 0.394) chi-square relationship was found between the SES categories in the control and AD groups. No significant difference was seen between variables of the control and AD groups in subjects of different SES. Thus, we can state that variations in the SES of patients with AD do not affect the parameters.

## Discussion

Many studies have reported that high levels of glucocorticoid receptors are located in the hippocampus, where they are engaged in the negative feedback of glucocorticoid secretion.^14^ Since degeneration of the hippocampus is a prominent characteristic of AD, the loss of hippocampal cells leads to hypercortisolaemia in AD. This in turn enhances the degeneration of the hippocampus as the disease progresses. A study by Dhikav et al., presented that the cortisol levels of patients with AD were higher and yet were within normal limits.^4^ However, in our study, the serum levels of cortisol in patients with AD were not within the normal range. The serum levels of cortisol in patients with AD were significantly higher than those in the control group.

Recent studies have shown that hyperhomocysteinemia leads to hippocampal degeneration in the brains of patients with AD.^15^ The results of a prospective, observational study by Sudha Seshadri et al., indicated that there was a strong, graded association between plasma Hcy levels and the risk of AD. According to their results, plasma homocysteine levels greater than 14 μmol/l doubles the risk of AD occurrence. We found that the serum levels of Hcy were significantly increased in patients with AD, as compared with healthy controls. These results are in agreement with those of Refsum et al., and others.^16-18^

There are various mechanisms by which hyperhomocysteinemia can contribute to neuronal degeneration. Elevated Hcy may activate the N-methyl-D-aspartate receptors that cause cell death or also might get transformed into homocysteic acid, thereby exerting its harmful neurotoxic effects.^18^ Neuroinflammation is known to be an important feature of AD and has also been recognised to be linked with chronic stress-related disorders. The effects of chronic stress and hyperhomocysteinemia on inflammatory cytokines are complex in distinct diseases.^5,19^ In our study, we found that the serum IL-6 levels were significantly increased in patients with AD, as compared with controls. Our results coincide with the findings of several studies, such as those of Kim and many others.^19-21^ Yu San Chang et al., found higher levels of cortisol and a decline in global cognition in patients with AD as compared with controls.^14^ Similarly, in our study, we found that the cognitive functions of patients with AD, as shown by the MMSE scores, were significantly decreased.

A study by John G. Csernansky et al., have shown that, in subjects with dementia, there is a strong association between higher plasma cortisol levels and rapidly increasing symptoms of dementia. This is related to the rapidly decreasing performance of patients on neuropsychological tests associated with temporal lobe function. They concluded that enhanced HPA activity, as reflected by hypercortisolaemia, is associated with more rapid disease progression in patients with AD.^22^ In line with these findings, our results showed a significant negative correlation between the serum cortisol levels and MMSE scores in AD. In AD, the MMSE scores were also strongly negatively correlated with the serum levels of IL-6 and Hcy. Our results agree with those of Grzegorz Raszewski et al., who showed a significant relationship between MMSE scores and serum levels of total Hcy in dementia.^23^ The serum levels of cortisol and homocysteine showed a significant positive relationship in the AD group. The current guidelines for AD diagnosis require the discovery of potential biomarkers.^24^ In their study, Hasselgren et al.,^11^ stated that gender and SES have a great impact on AD conditions. However, we did not find any variations in the study parameters of AD subjects with regards to gender or SES. This study comprised some limitations, such as a valid but small sample size due to insufficient funding. Incomprehension in societies about the gravity of AD severity and consequent delay by family members in seeking medical attention for AD patients results in catastrophic lack of access to care for the patients.

In this study, it can be concluded that the cognitive decline in AD was found to be strongly associated with hypercortisolaemia and hyperhomocysteinemia, together with inflammation. Further molecular research involving correlations of stress and inflammatory markers would be beneficial to discover reliable biomarkers that would complement clinical tests in the early and accurate diagnosis of AD.

## Figures and Tables

**Figure 1.**
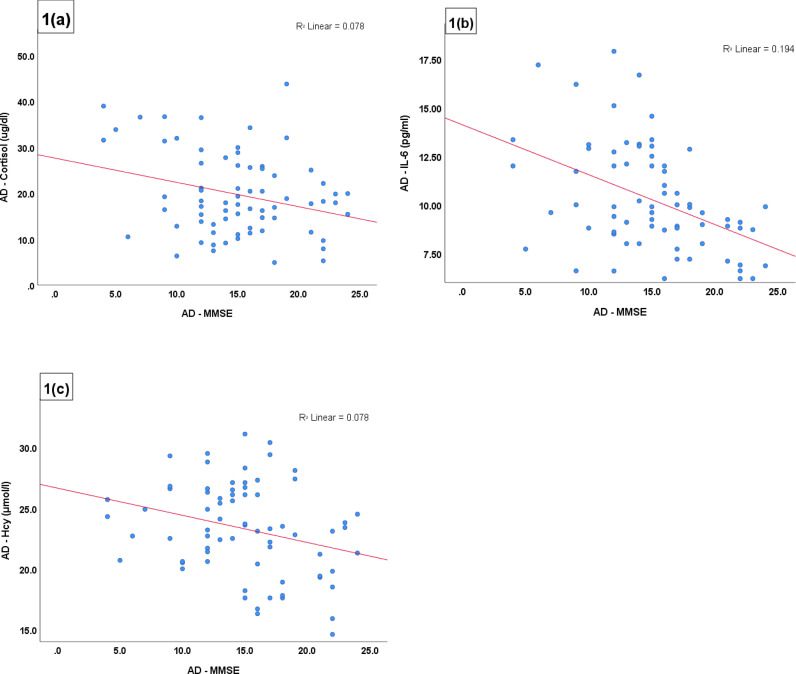
Correlations between parameters in the AD group (a) MMSE score and cortisol, (b) MMSE score and IL-6 (c) MMSE and Hcy

**Table 1:** Age, MMSE, cortisol, IL-6 and homocysteine in the control and AD groups^*^

Variables	Control (n=70)	AD (n=71)	Mean difference	Std. error difference	t	*p*-value

Age (years)	68.65 ± 5.98	70 ± 7.02	–1.343	1.099	–1.221	0.224

MMSE	24.7 ± 3.06	14.97 ± 4.74	9.728	0.671	14.499^**HS**^	< 0.001

Cortisol (ug/dl)	13.373 ± 5.587	19.697 ± 8.956	–6.324	1.255	–5.038^**HS**^	< 0.001

IL-6 (pg/ml)	3.371 ± 0.798	10.272 ± 2.762	–6.901	0.341	–20.217^**HS**^	< 0.001

Hcy (μmol/l)	8.249 ± 2.358	23.292 ± 3.806	–15.043	0.532	–28.254^**HS**^	< 0.001


^*^AD: Alzheimer's disease; n: number of subjects; IL-6: Interleukin 6, Hcy: homocysteine, MMSE: Mini-Mental State Examination^HS^Student's t-test with *p* < 0.01 is statistically highly significant.

**Table 2:** Distribution of variables within the control and AD groups according to gender^*^

Groups	Variables	Males	Females	U^†^	*p*-value

Control	Age (years)	70.13 ± 5.59	66.70 ± 6.02	370.5^**HS**^	0.006

group	MMSE	24.70 ± 3.19	24.70 ± 2.93	592.5	0.929

(n = 71)	Cortisol (ug/dl)	13.09 ± 5.00	13.74 ± 6.36	581.00	0.822

	IL-6 (pg/ml)	3.29 ± 0.77	3.49 ± 0.83	517.5	0.327

	Hcy (μmol/l)	8.56 ± 2.52	7.83 ± 2.09	522.5	0.358

AD	Age (years)	71.20 ± 7.52	67.80 ± 5.47	422.00	0.065

group	MMSE	14.63 ± 5.54	15.60 ± 2.72	500.50	0.368

(n = 70)	Cortisol (ug/dl)	20.14 ± 9.11	18.88 ± 8.79	525.00	0.547

	IL-6 (pg/ml)	10.04 ± 2.93	10.70 ± 2.42	465.00	0.185

	Hcy (μmol/l)	23.65 ± 4.15	22.64 ± 3.03	474.00	0.224


^*^AD: Alzheimer's disease; n: number of subjects; IL-6: Interleukin-6; Hcy: homocysteine; MMSE: Mini-Mental State Examination^†^Mann-Whitney U test applied to estimate the difference between variables in males and females of the control and AD groups.^HS^Highly significant at *p*>0.01.

**Table 3:** MMSE, cortisol, IL-6 and homocysteine in control and AD subjects according to SES^*^

Parameters	Upper class	Upper middle class	Lower middle class	Upper lower class	Lower class	*p*-value^†^

Control group						

n (%)	6 (8.45)	18 (25.35)	21 (29.58)	15 (21.13)	10 (1.41)	–

MMSE	25.83 ± 1.60	25.72 ± 2.95	24.71 ± 3.13	23.53 ± 3.50	23.90 ± 2.64	0.233

Cortisol (ug/dl)	13.61 ± 6.41	13.29 ± 4.79	12.32 ± 6.02	12.40 ± 4.77	17.05 ± 6.17	0.238

IL-6 (pg/ml)	3.57 ± 0.65	3.31 ± 0.75	3.43 ± 0.85	3.17 ± 0.84	3.53 ± 0.87	0.762

Hcy (μmol/l)	8.20 ± 3.89	8.62 ± 2.28	7.96 ± 2.26	7.68 ± 2.17	9.07 ± 1.98	0.593

AD group						

n (%)	2 (2.85)	5 (7.14)	32 (45.71)	28 (40)	4 (5.71)	–

MMSE	14.00 ± 2.83	13.2 ± 4.26	15.56 ± 4.99	14.64 ± 4.91	15.25 ± 3.59	0.848

Cortisol (ug/dl)	24.77 ± 13.34	27.06 ± 9.09	19.97 ± 8.81	18.81 ± 8.48	11.96 ± 7.54	0.117

IL-6 (pg/ml)	9.99 ± 0.84	10.34 ± 2.57	10.18 ± 3.03	10.29 ± 2.78	10.87 ± 1.91	0.994

Hcy (μmol/l)	25.25 ± 2.89	26.7 ± 2.58	22.64 ± 3.85	23.44 ± 3.55	22.23 ± 5.61	0.212


^*^AD: Alzheimer's disease; IL-6: Interleukin-6; Hcy: homocysteine; MMSE: Mini-Mental State Examination; SES: socioeconomic status; n (%): number of subjects (percentage)^†^ANOVA test for differences between variables in SES categories of the AD group.

**Table 4:** Correlations between MMSE, cortisol, Hcy and IL-6 between the control and AD groups^*^

Control group/AD group	r-value	*p*-value	Control group/AD group	r-value	*p*-value

C-MMSE/AD-MMSE	–0.047	0.702	C-MMSE/AD-IL-6	0.015	0.899

C-Cortisol/AD-Cortisol	–0.224	0.063	C-Cortisol/AD-MMSE	0.208	0.084

C-Hcy/AD-Hcy	–0.162	0.180	C-Cortisol/AD-IL-6	–0.235	0.050

C-IL-6/AD-IL-6	–0.038	0.758	C-Hcy/AD-MMSE	0.251^S^	0.036

C-MMSE/AD-Cortisol	–0.054	0.656	C-Hcy/AD-Cortisol	–0.266^**S**^	0.026

C-MMSE/AD-Hcy	0.098	0.420	C-IL-6/AD-MMSE	0.112	0.354


^*^AD: Alzheimer's disease; C: control; IL-6: Interleukin-6; Hcy: homocysteine; MMSE: Mini-Mental State Examination;^S^Pearson's coefficient *p* < 0.05 is statistically significant.
